# Prevalence of Severe Acute Respiratory Syndrome Coronavirus 2 Neutralizing Antibodies in Egyptian Convalescent Plasma Donors

**DOI:** 10.3389/fmicb.2020.596851

**Published:** 2020-11-24

**Authors:** Mokhtar R. Gomaa, Ahmed Kandeil, Ahmed Mostafa, Wael H. Roshdy, Ahmed E. Kayed, Mahmoud Shehata, Omnia Kutkat, Yassmin Moatasim, Ahmed El Taweel, Sara H. Mahmoud, Mina Nabil Kamel, Noura M. Abo Shama, Mohamed El Sayes, Rabeh El-Shesheny, Osama H. Bakheet, Mohamed A. Elgohary, Mohamed Elbadry, Naguib N. Nassif, Salwa H. Ahmed, Ibrahim Y. Abdel Messih, Ghazi Kayali, Mohamed A. Ali

**Affiliations:** ^1^Center of Scientific Excellence for Influenza Viruses, National Research Center, Giza, Egypt; ^2^Central Public Health Laboratory, Ministry of Health and Population, Cairo, Egypt; ^3^Department of Clinical Pathology, Military Medical Academy, Cairo, Egypt; ^4^Department of Tropical Medicine, Cairo University, Cairo, Egypt; ^5^Tropical Medicine and Gastroenterology Department, Aswan University, Aswan, Egypt; ^6^Preventive Medicine Department, Aswan Health Affairs Directorate, Aswan, Egypt; ^7^Clinical Pharmacology Department, Aswan Fever Hospital, Aswan, Egypt; ^8^Faculty of Medicine, Ain Shams University, Cairo, Egypt; ^9^Department of Epidemiology, Human Genetics, and Environmental Sciences, University of Texas, Houston, Texas, TX, United States; ^10^Human Link, Baabda, Lebanon

**Keywords:** severe acute respiratory syndrome coronavirus 2, Egypt, plasma donors, neutralizing antibodies, microneutralization assay

## Abstract

Using convalescent plasma as immunotherapy is an old method for treatment of infectious diseases. Several countries have recently allowed the use of such therapy for the treatment of COVID-19 patients especially those who are critically ill. A similar program is currently being tested in Egypt. Here, we tested 227 plasma samples from convalescent donors in Egypt for neutralizing antibodies against severe acute respiratory syndrome coronavirus 2 (SARS-CoV-2) using a microneutralization (MN) assay. A third of the tested samples did not have antibody titers and 58% had titers between 1:10 and 1:80. Only 12% had titers >1:160. We also compared MN assays using different virus concentrations, plaque reduction neutralization (PRNT) assays, and a chemiluminescence assay that measures immunoglobulin G (IgG) binding to N and S proteins of SARS-CoV-2. Our results indicated that a MN assay using 100 TCID50/ml provides comparable results to PRNT and allows for high throughput testing.

## Introduction

According to the World Health Organization’s (WHO) COVID-19 situation report number 181, the world has reported almost 14 million cases and 6,00,000 deaths (4.2% case fatality rate; WHO, 2020a). This disease, caused by the Severe Acute Respiratory Syndrome Coronavirus 2 (SARS-CoV-2), was first reported in China in December 2019. Following that, the virus spread globally causing epidemics in almost every country. As with other infectious diseases, an effective vaccine would be the best weapon to slow the spread of the virus but this is not yet available. According to the WHO, there are currently 10 candidate vaccines in clinical evaluation while 126 others are in pre-clinical phases (WHO, 2020b). Until a vaccine is proven effective, produced, and distributed, interventions to reduce transmission focus on social and physical distancing, hand hygiene, using masks, and other personal protective equipment. There is no SARS-CoV-2 specific antiviral treatment, so the management of COVID-19 cases focuses mainly on treating symptoms. However, several therapeutics are currently being tested through the WHO’s “Solidarity” trial and other trials including remdesivir, chloroquine and hydroxychloroquine, lopinavir and ritonavir, and lopinavir + ritonavir + interferon-beta (Brown and Mccullough, 2020).

Passive immune therapy has been employed in the treatment of several infectious diseases including COVID-19. In the United States, the Food and Drug Administration (FDA) approved the use of convalescent plasma to treat critically ill patients (Tanne, 2020). Among 25 patients who received this treatment, 76% showed clinical improvement and none showed adverse events (Salazar et al., 2020). A critically ill obstetrics patient also improved after receiving convalescent plasma therapy (Anderson et al., 2020). Two patients in South Korea who received convalescent plasma improved (Ahn et al., 2020). In a case series of eight patients from Iran, authors reported improved respiratory status and total recovery among seven of the patients (Adeli et al., 2020). Similarly, several reports from China cited improvement upon receiving convalescent plasma (Duan et al., 2020; Kong et al., 2020; Shen et al., 2020).

Egypt, like almost any other country, reported cases and deaths of COVID-19. The first case was reported on 6 March 2020 and as of 15 June 2020. Egypt had more than 42,000 cases and 1,672 deaths (3.6% case fatality rate). Studying the effectiveness of convalescent plasma therapy is ongoing. To this end, we tested plasma from convalescent donors for antibodies against SARS-CoV-2. We also compared various protocols for testing neutralizing antibodies.

## Materials and Methods

### Plasma

RT-PCR-confirmed convalescent adults (*n* = 227) who have been symptom-free for at least 2 weeks donated plasma as part of routine blood and blood products donation programs at the Military Medical Academy, Ain Shams University Hospitals, Egypt. Three human serum samples, collected during the flu season 2018–2019, were used as a negative control. Positive sera from mice immunized with an inactivated SARS-CoV-2 vaccine were used as positive controls in microneutralization (MN) and plaque reduction neutralization (PRNT) assays. This study was approved by the Ethics Committee of the National Research Center. Plasma was processed for testing at the same day of receipt in the lab. All samples were heat-inactivated at 56°C for 30 min and stored at −20°C before testing.

### Plaque Infectivity Assay

For the titration of the different tissue culture infectious dose 50 (TCID50) of human coronavirus 2019 (hCoV-19)/Egypt/NRC-03/2020 (hCoV-19_NRC-03, Accession Number in GSAID database: EPI_ISL_430820), plaque infectivity assay was carried out. Briefly, the 200, 100, and 50 TCID50/ml of hCoV-19_NRC-03 were serially diluted 10-fold in FBS-free media then 100 μl of each dilution was mixed with 200 μl of infection medium and used to inoculate 80–90% confluent Vero E6 cells into one well of a 6-well plate. A control well was included in the plate that was inoculated with 300 μl of serum free medium. The plate was incubated at 37°C under 5% CO_2_ for 1 h to allow virus adsorption and rocked every l5 min to ensure homogenous exposure of the cells to infection and avoid drying of cells. After 1 h, 3 ml of the over layer medium were added, and the plate was agitated to allow homogenous mixing of the virus inoculum through the over layer. To allow the solidification of the agarose component of the over layer medium, the plate was left at room temperature for about 10 min then further incubated at 37°C under 5% CO_2_. After 72 h, 1 ml of fixation solution was added to each well for 1 h for cell fixation and virus inactivation. The fixer was later discarded, and the plate wells were flushed with water and dried. For visualization of the plaques, 1 ml of the staining solution (0.1% crystal violet) was added to each well for 5 min, the dye was discarded, and the plate wells were rinsed with water and dried. Viral plaques were visualized as clear unstained spots (due to viral infection) in a violet (stained cells) background. The virus titer was calculated through the following equation:

Plaque forming unit (PFU)/ml = Number of plaques × inoculated volume of the virus × virus dilution ×10.

### Microneutralization Assay

The MN is conducted as described previously using Vero-E6 cell monolayers (Perera et al., 2013). Briefly, serial 2-fold dilutions of heat-inactivated plasma starting with a dilution of 1:10 were mixed with equal volumes of 200, 100, and 50 TCID50/ml of hCoV-19/Egypt/NRC-03/2020 SARS-CoV-2 isolate. After 1 h of incubation at 37°C, 35 μl of the virus-plasma mixture was added in duplicate to Vero-E6 cell monolayers in 96-well microtiter plates. After 1 h of adsorption, the inoculums were aspirated. The plates were then incubated for 3 more days at 37°C in 5% CO_2_ in a humidified incubator. A virus back-titration was performed without immune serum to assess input virus dose. Cytopathic effect (CPE) was read at 3 days post infection. The highest serum dilution that completely protected the cells from CPE was recorded as the neutralizing antibody titer.

### Plaque Reduction Neutralization Assay

Vero-E6 cells were seeded in 12-well culture plates (10^5^ cells/ml) and incubated for 24 h at 37°C in 5% CO_2_. Previously titrated hCoV-19/Egypt/NRC-03/2020 SARS-CoV-2 isolate was diluted to the optimal virus dilution that gave countable plaques (10^−2^) and mixed with the heat-inactivated plasma. The virus-plasma mixtures were incubated for 1 h at 37°C before being added to cells. Growth medium was removed from the 12-well cell culture plates, and virus-sera mixtures were added to cells. After 1 h contact time for virus adsorption, 3 ml of DMEM supplemented with 2% agarose, 1% antibiotic antimycotic mixture, and 4% bovine serum albumin were added to the cell monolayer. The plates were left to solidify and incubated at 37°C until the formation of viral plaques (3 days). Formalin (10%) was added to each well for 1 h and the overlayer removed. Fixed cells were stained with 0.1% crystal violet in distilled water. Untreated virus was included in each plate as control. Plaques were counted and the dilution of sera that gave 50% inhibition was counted. The neutralization titer (PRNT50) of the test plasma sample is defined as the reciprocal of the highest dilution of plasma that reduces 50% of counted plaques compared with the untreated virus.

### Chemiluminescence Assay

Immunoglobulin G (IgG) against SARS-CoV-2 in plasma samples were tested using iFlash 1800 (YHLO Biotech, Shenzhen, China) according to the manufacturer’s instructions. N and S antigens were coated on magnetic beads, and 200 μl of plasma were added undiluted. After antigen-antibody binding, anti-human IgG was added.

### Statistical Analysis

Wilcoxon rank sum test was used to compare titers. Spearman’s rho coefficient was used to assess correlation between titers. Statistical significance was determined at values of *p* ≤ 0.05. IBM SPSS Statistics software version 23 (IBM, Armonk, NY, United States) was used.

## Results

### Assay Comparisons

**Figure 1:**
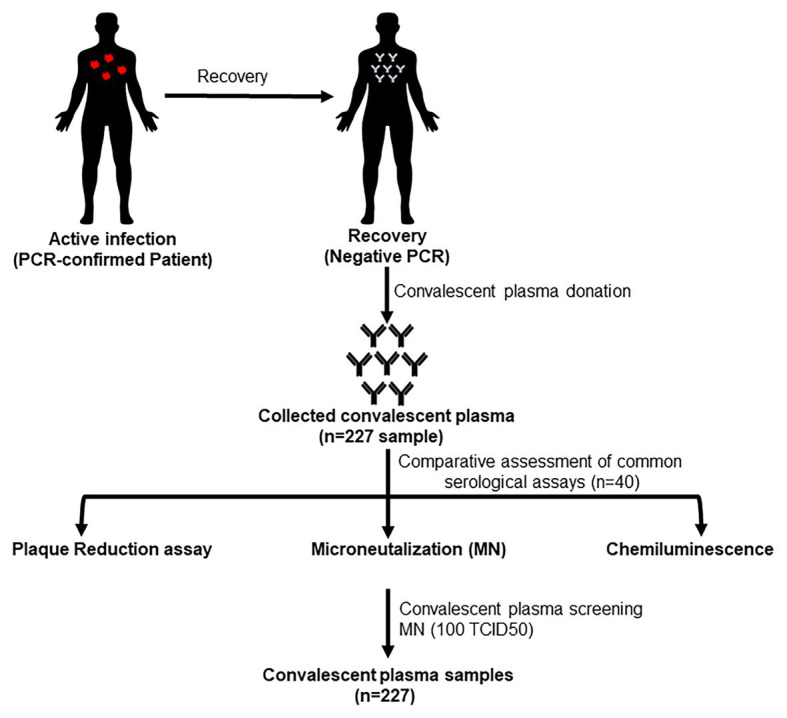
Scheme showing convalescent plasma donors who were involved in the study.

**Figure 2:**
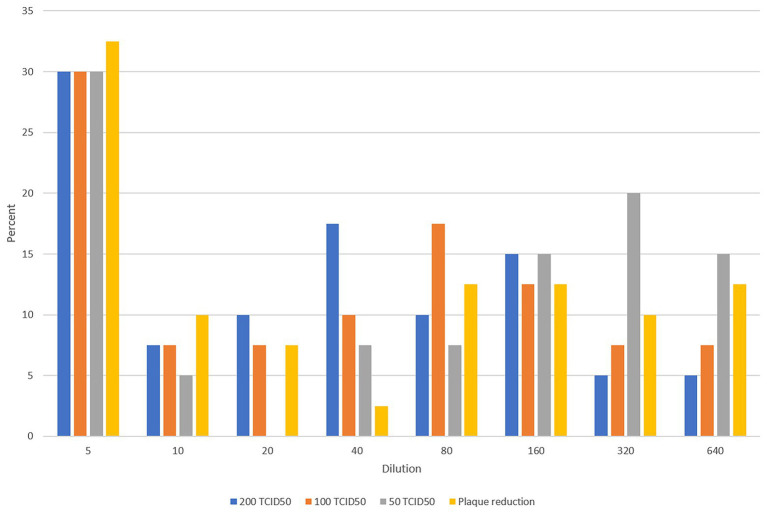
Distribution of titers resulting from various neutralization assays.

Figure 1 shows the testing scheme followed in this study. We initially investigated potential assays to test the sera to determine antibody levels and suitability for use as convalescent plasma therapy in comparison to PRNT which is considered the golden standard for measuring neutralizing antibody titers. The first set of 40 plasma samples were tested by PRNT, chemiluminescence, and MN using 200, 100, and 50 TCID50/ml virus dilutions. To translate the applied TCID50/ml titers into PFU/ml, the three applied TCID50/ml were titrated using plaque infectivity assay (Table 1). The geometric mean titers (GMTs) and titer ranges are shown in Table 1. GMTs for the MN assay ranged between 30.3 and 59.6 increasing as the virus concentration decreased. The GMT for PRNT was 38.0 falling between the GMTs calculated for the 100 and 50 TCID50/ml MN GMTs. The GMT calculated for the chemiluminescence assay was 9.1. The titers for all neutralizing assays were 5–640 while that for chemiluminescence was 0.1–135. The distribution of titers generated by the neutralizing assays is shown in Figure 2. The 50 TCID50/ml MN assay provided higher proportions of sera as having higher titers than measured by the other assays. All negative and positive sera controls yielded the expected result in all assays.

**Table 1:** Geometric means and ranges of titers resulting from various neutralization assays.

Assay	TCID50	FFU/ml	Geometric mean titer	Titer range
MN	200	1.9 × 10^6^	30.3	5–640
MN	100	9.9 × 10^5^	35.4	5–640
MN	50	5 × 10^5^	59.6	5–640
PRNT	100	9.9 × 10^5^	38.0	5–640
Chemiluminescence	ND	ND	9.1	0.1–135

ND, not defined.

Measured titers were compared as ordinal variables using the Wilcoxon rank sum test and the resulting *p* values are shown in Table 2. Titers significantly differed between the three different dilutions used for MN assays. Results from the PRNT assay were significantly different than the 200 and 50 TCID50/ml MN assays but were not different from the 100 TCID50/ml MN. Titers generated using chemiluminescence differed significantly than all neutralizing assays.

**Table 2:** *p* values of Wilcoxon Sum Rank Test comparisons of various assays.

	200 TCID50	100 TCID50	50 TCID50	PRNT	Chemiluminescence
200 TCID50	–	NS	<0·01	0·049	0.045
100 TCID50		–	<0·01	NS	0.02
50 TCID50			–	0·014	<0·01
PRNT				–	0.02
Chemiluminescence					–

NS, not statistically significant.

Spearman correlation coefficients are shown in Table 3. There were strong correlations between the three MN assays (rho 0.87–0.95). The PRNT assay had strong correlations with all three MN assays (rho 0.79–0.85). The chemiluminescence assay showed weak correlation with the MN and PRNT assays (rho ≤0.26). Correlation scatterplots are shown in Supplementary Figure 1.

**Table 3:** Spearman correlation coefficients and values of *p* resulting from comparisons of various assays.

	200 TCID50	100 TCID50	50 TCID50	PRNT	Chemiluminescence
200 TCID50	–	0·95(<0·01)	0·87(<0·01)	0·79(<0·01)	0·19(NS)
100 TCID50		–	0·92(<0·01)	0·85(<0·01)	0·2(NS)
50 TCID50			–	0·85(<0·01)	0·19(NS)
PRNT				–	0·26(NS)
Chemiluminescence					–

### Distribution of Neutralizing Antibodies in Plasma

**Figure 3:**
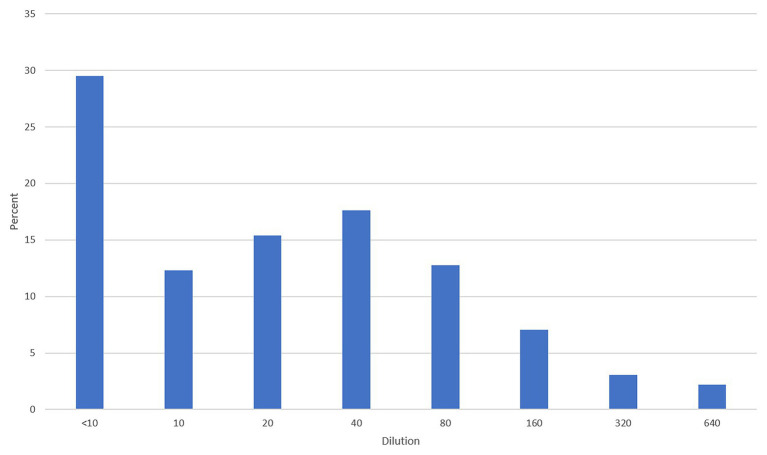
Distribution of titers in all tested plasma samples using the 100 TCID50/ml microneutralization (MN) assay.

All plasma samples were tested by MN with 100 TCID50/ml. The GMT was 22.4, and the range was 5–640. The distribution of titers is shown in Figure 3. A third of the tested samples did not have neutralizing antibodies. Around 58% of the samples had antibody titers ranging from 1:10 to 1:80, while around 12% had titers of 1:160 and higher.

## Discussion

Several countries reported using convalescent plasma as part of therapeutic intervention against COVID-19 especially for critically ill patients. The efficacy of using this strategy remains uncertain. Although several countries reported some improvement in plasma-treated patients, the evidence remains observational and not corroborated by findings of randomized clinical trials (RCTs). A review article that included five studies among 27 patients noted an overall improvement in outcomes (Rajendran et al., 2020). A Chinese-based RCT among 103 patients found no significant differences between patients receiving plasma as compared to the control group. However, this study was terminated early and may have not achieved the sample size required to draw final conclusions (Li et al., 2020). Hence, more RCTs are needed to conclude whether plasmapheresis is an effective treatment for COVID-19 or not.

Protocols for using convalescent plasma therapy for COVID-19 are not yet standardized. However, most protocols require that patients should be convalescent for 2–4 weeks prior to donation, that only adults donate, that routine procedures for accepting blood and blood products donations be followed, and that plasma is tested to quantitatively assess the levels of antibodies (Chen and Xia, 2020; Tiberghien, 2020). Antibody titer thresholds are also not standardized but a titer of 1:160 of SARS-CoV-2 specific IgG is recommended (Chen and Xia, 2020).

The Egyptian public health authorities are testing the effectiveness of convalescent plasma therapy for COVID-19 patients. Collected plasma was tested for neutralizing antibodies using MN assays with various virus concentrations as well PRNT, which is considered the golden standard for measuring neutralizing antibodies. Although the assays correlated well with each other, differences in titer levels were noted as titers increased when the virus concentration decreased. Results of testing sera with 100 TCID50/ml MN assay did not differ from PRNT results. Hence, we recommend using MN with 100 TCID50/ml for testing plasma as it has comparable results and strong correlation with PRNT and allows high throughput testing.

There is a strong correlation between immunoglobulin titers against the spike (S) protein of SARS-CoV-2 and neutralizing antibodies titers (Amanat et al., 2020). We also compared neutralizing antibody assays to chemiluminescence that assesses IgG binding to SARS-CoV-2 N and S proteins. Weak correlation was noted between chemiluminescence and all neutralizing antibody assays, and significant differences in titers were noted. The chemiluminescence assay gave a lower maximum titer and a lower GMT. Hence, although this assay may be sensitive and specific, its accuracy is questionable. We recommend testing plasma for neutralizing antibodies or using assays that have been extensively validated for sensitivity, specificity, and accuracy such as the ELISA protocol developed previously (Amanat et al., 2020).

Our data indicate that not all convalescent plasma have antibodies against SARS-CoV-2 as the third of the tested samples were negative. Furthermore, around 58% had antibody titers between 1:10 and 1:80 that may make the plasma not useful as therapy. Only a small percentage had high antibody titers ≥1:160. A similar study in England revealed that around a quarter of the donors did not have neutralizing antibodies but had relatively higher titers than our study (Harvala et al., 2020).

A main limitation of this study was that information on the donor population was not available, and plasma samples were only provided with blind codes. Therefore, we were not able to correlate the results to gender, age, or the health status of the donors. Clinical data were not accessible to correlate with seroconversion and titers.

In conclusion, it is important to test the plasma for the presence of antibodies prior to administration as a therapeutic. Large serological studies among known recovered patients and in the general populations are needed to understand the true frequencies, levels, determinants, and durations of seroconversion and seropositivity.

## Data Availability Statement

The original contributions presented in the study are included in the article/Supplementary Material, further inquiries can be directed to the corresponding authors.

## Ethics Statement

The studies involving human participants were reviewed and approved by Ethics Committee of the National Research Centre. The patients/participants provided their written informed consent to participate in this study.

## Author Contributions

Conceptualization, formal and analysis, and writing — review and editing: AK, AM, GK, and MAA. Methodology: AK, AM, MRG, GK, and MAA. Investigation: MRG, AM, AK, WHR, AEK, MS, OK, YM, AET, SHM, MNK, NMA, MES, RE-S, OHB, MAE, ME, NNN, SHA, and IYA. Data curation: AK and AM. Writing — original draft preparation: AK, AM, and GK. Supervision and funding acquisition: GK and MAA. All authors have read and agreed to the published version of the manuscript.

### Conflict of Interest

The authors declare that the research was conducted in the absence of any commercial or financial relationships that could be construed as a potential conflict of interest.
