# Supplementary figures and images for: Prevalence of Severe Acute Respiratory Syndrome Coronavirus 2 Neutralizing Antibodies in Egyptian Convalescent Plasma Donors
Source: Front Microbiol. 2020 Nov 24;11:596851. doi: 10.3389/fmicb.2020.596851 (PMC7732494; doi:10.3389/fmicb.2020.596851)

Figure S: Correlation between titers generated from various assays

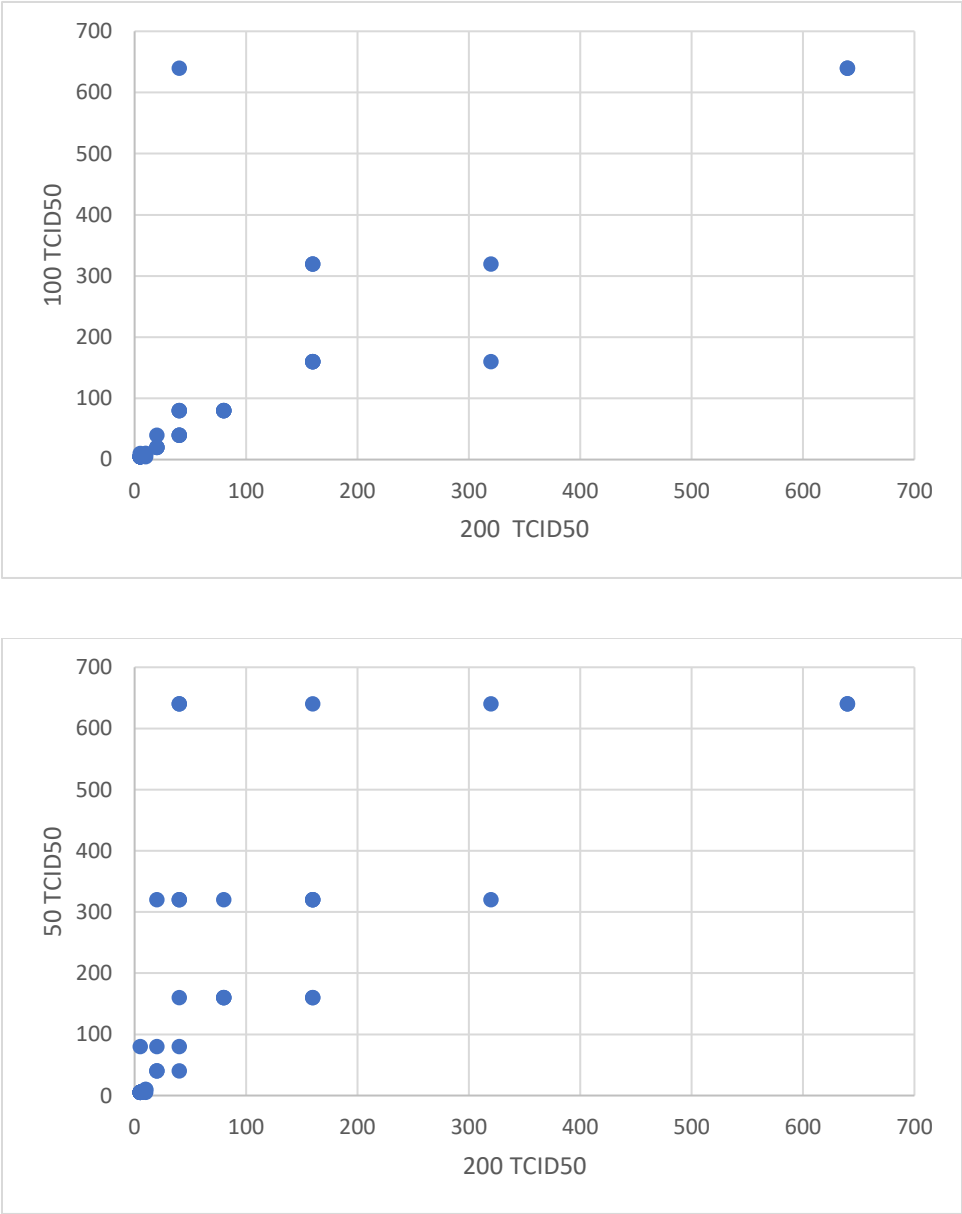

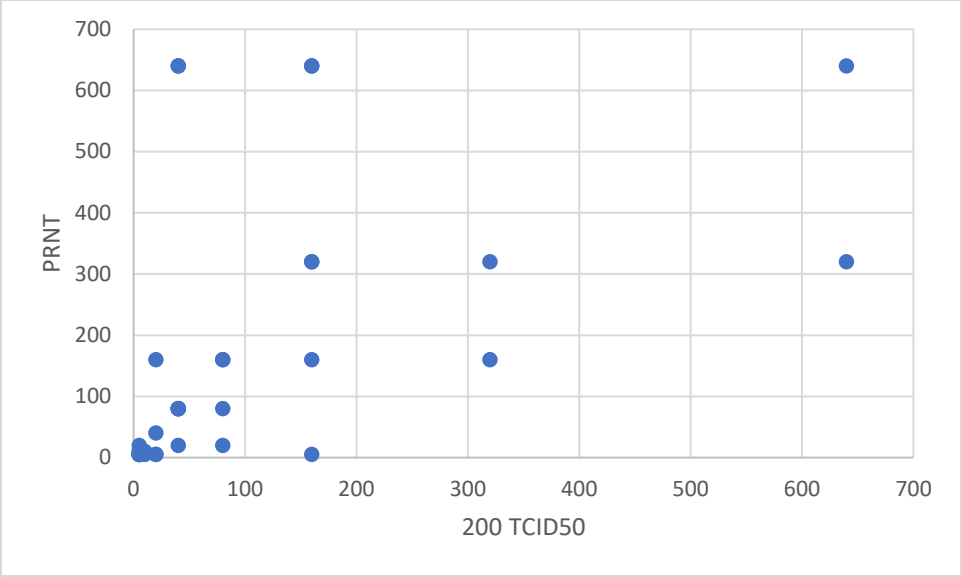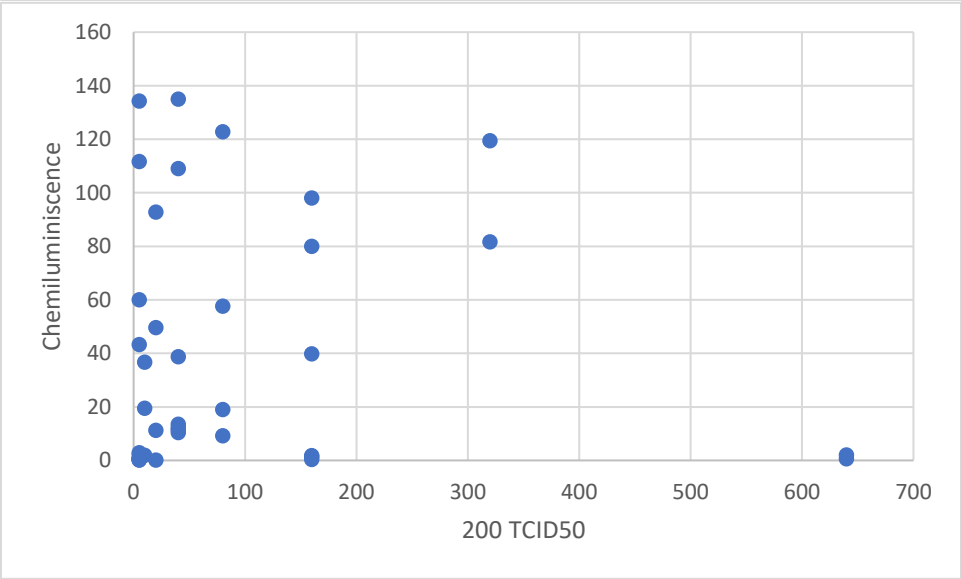

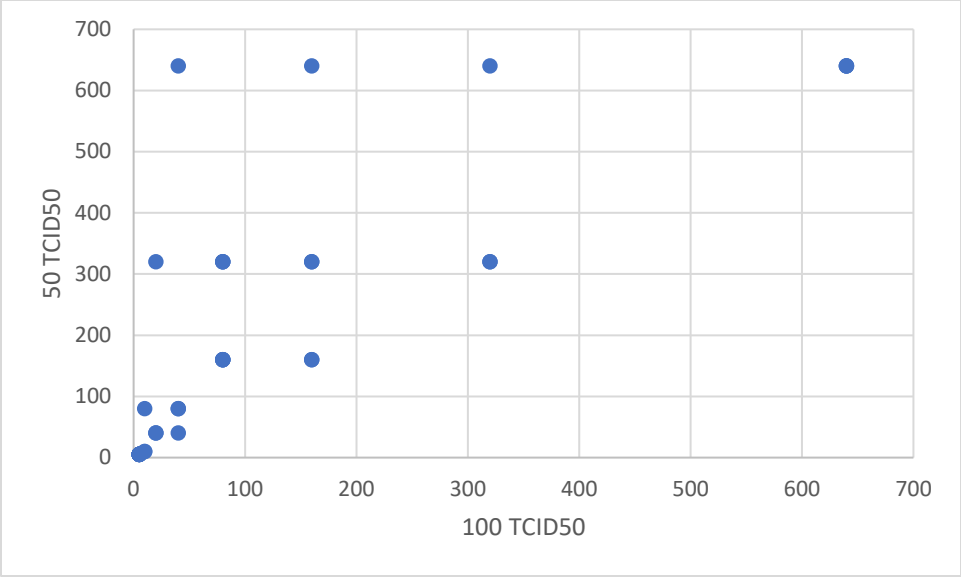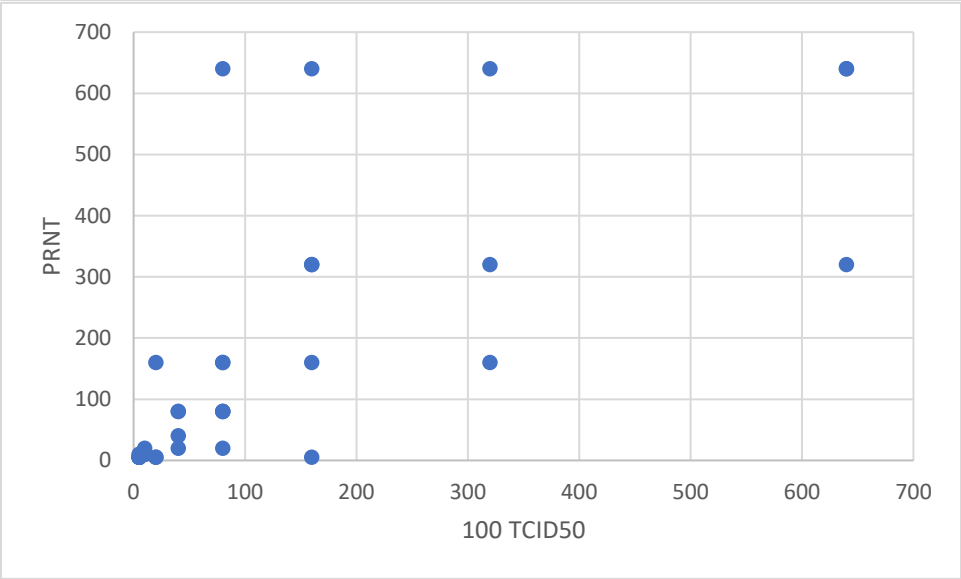

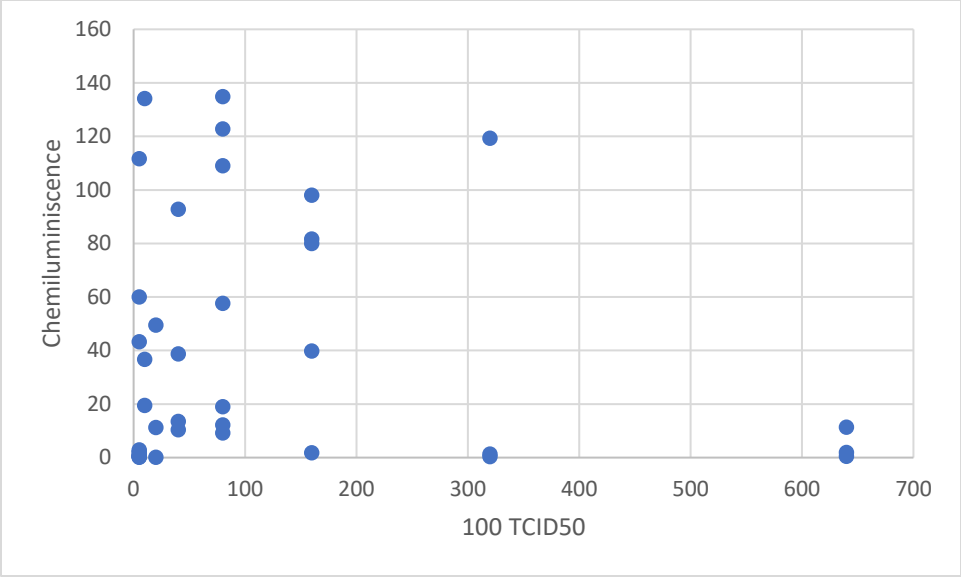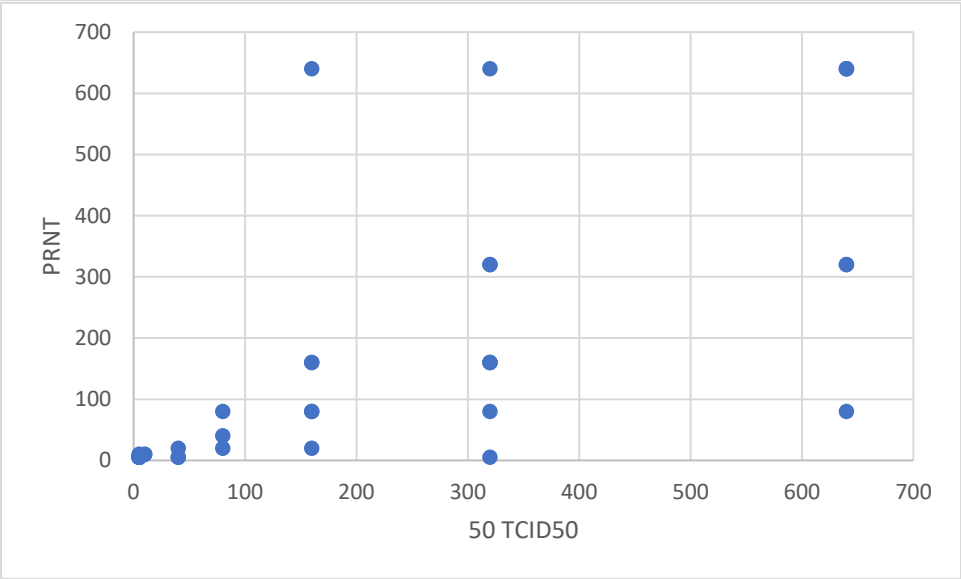

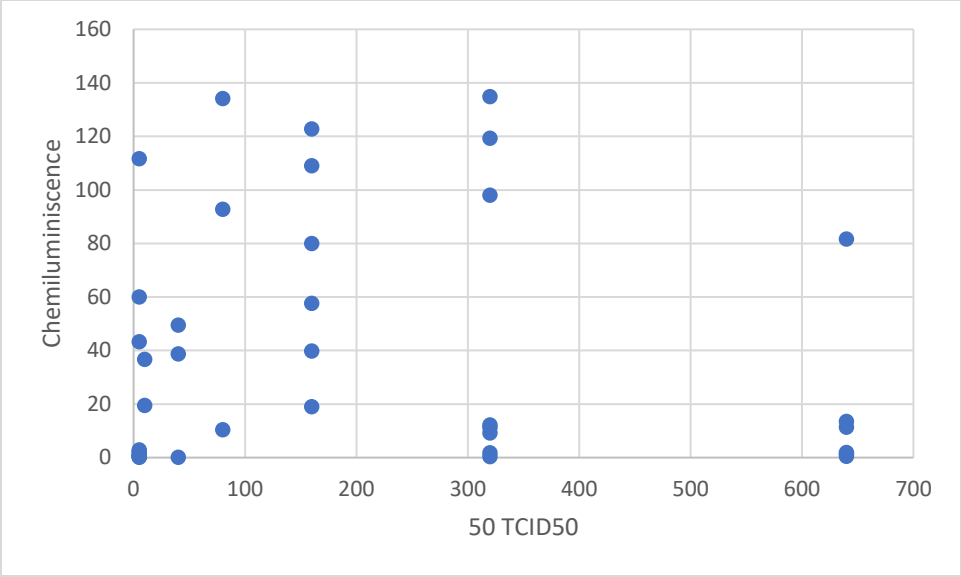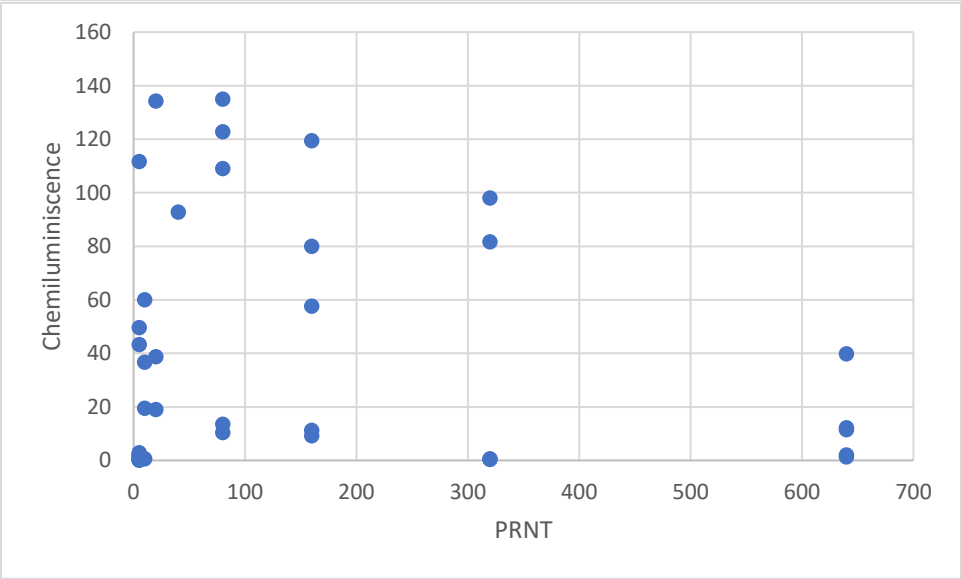

Supplement: Supplementary file 1 [file Data_Sheet_1.pdf]
